# Minimally Invasive Rehabilitation of Posterior Erosive Tooth Wear: Two Case Reports of the One-Stage Dahl Approach

**DOI:** 10.7759/cureus.22235

**Published:** 2022-02-15

**Authors:** In Meei Tew, Edward Huen Tai Ho

**Affiliations:** 1 Restorative Dentistry, The National University of Malaysia, Kuala Lumpur, MYS; 2 Oral Rehabilitation, The University of Hong Kong, Hong Kong, CHN

**Keywords:** indirect restoration, direct restoration, conservative treatment, erosive tooth wear, dahl concept

## Abstract

Rehabilitation of posterior erosive tooth wear can be especially challenging in the presence of substantial tooth structure loss and limited inter-occlusal space. This article describes two case reports illustrating a conservative approach using the one-step Dahl approach in the management of localized posterior erosive tooth wear. The occlusal surfaces of worn teeth in both cases were successfully restored using direct composite resin and gold onlay, respectively. The material was placed in supra-occlusion during the initial stage. This technique enables intrusion of affected worn teeth and the opposing counterpart. Eruption of the remaining dentition will occur after two to three months to re-establish a complete occlusal relationship. With proper treatment planning, the one-step Dahl approach offers a simpler and predictable positive outcome in restoring structurally compromised posterior worn teeth.

## Introduction

Dental erosion leads to irreversible tooth surface loss. The etiology could be due to intrinsic (acid reflux and frequent vomiting) and/or extrinsic factors (dietary habits, ingestion of acidic foods and beverages, industrial and environmental chemicals) [1]. Although dental erosion is more commonly found on the palatal surfaces of the anterior teeth, the occlusal surfaces of mandibular molars can also be frequently affected [2].

Rehabilitation of posterior erosive worn tooth can be challenging if the affected tooth suffers substantial tooth structure loss and is accompanied by dentoalveolar compensation, which limits the inter-occlusal spaces [3]. Traditionally, a localized posterior worn tooth is restored by means of affixing a full-coverage crown that conforms to the existing occlusion. However, this approach requires substantial occlusal reduction, which negatively impacts the existing short crown height, prostheses retention, and pulp vitality [4]. Another approach would be to increase the occlusal vertical dimension to provide adequate space for restoration Nevertheless, it involves a high cost and increases the risk of restorative complications on other unaffected dentition.

The Dahl approach is an alternative technique that can restore localized worn teeth in a more conservative way. This technique was first developed in the management of advanced localized anterior tooth wear with limited inter-occlusal space. This technique utilizes the principle of relative axial tooth movement in response to the placement of a removable “bite raising appliance” to gain adequate inter-occlusal clearance for definitive restorations on affected anterior worn teeth [5]. Thus, tooth preparation will be minimal, which, in turn, preserves its viability. This approach has been subsequently applied on posterior worn teeth with favorable results reported [6].

Although the Dahl appliance is initially a removable device, it has been subsequently modified and adapted into restorations that are locally fixed on affected worn teeth to eliminate poor patient compliance to the treatment [7]. With the advancement of adhesive dentistry, the use of adhesive restorations, such as onlays and composite resin, as fixed Dahl appliances have gained popularity due to the conservative tooth preparation approach without compromising the bonding strength to tooth surfaces [8].

 A two-stage Dahl procedure known for its good treatment outcome is commonly used with interim appliance placement to create an inter-occlusal space prior to final restoration construction. This technique has been further modified into a one-stage approach. It involves the direct placement of definitive restorations in supra-occlusion on affected worn dentition without an intermediate appliance. This simplified one-stage procedure saves treatment visits for patients and reduces the cost of treatment material. Comparable results have been reported with the one-stage Dahl procedure although admittedly limited information is available. These case reports describe conservative management of posterior erosive tooth wear using a direct composite resin and metal onlay, which applies the one-stage Dahl procedure.

## Case presentation

Case report 1

A 50-year-old female patient was referred to the Oral Rehabilitative Clinic, Prince Phillip Dental Hospital, The University of Hong Kong, for localized tooth wear management. She complained of sensitivity in the mandibular right teeth, which had worsened for the past month. This had caused her a lot of difficulty in chewing. Her past medical history was unremarkable. Notably, the patient admitted to frequent pickles consumption over the past 10 years. Informed consent was obtained from the patient prior to clinical examination and treatments.

**Figure 1:**
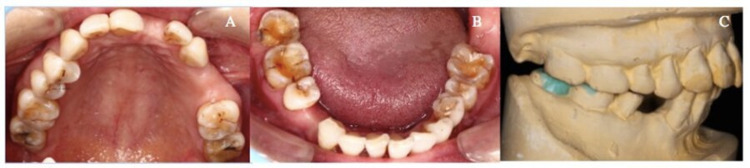
Preoperative photographs and diagnostic wax-up (A) Preoperative maxillary occlusal view. (B) Pre-operative mandibular occlusal view. (C) A diagnostic wax-up on a duplicated study cast at an increased occlusal vertical dimension of 1.5 mm.

The patient’s face was symmetrical with no temporomandibular joint abnormality. An intra-oral examination showed a missing maxillary left lateral incisor, first and second premolars, as well as a mandibular right second premolar (Figures 1A-1B). Signs of tooth surface loss were noted on the occlusal surface of the mandibular right first and second molars (grade III Smith and Knight Tooth Wear Index), as well as the mandibular left first molar (grade II Smith and Knight Tooth Wear Index) (Figure 1B). All teeth with tooth surface loss were responsive to pulp sensibility testing and appeared periodontally healthy. No radiographic evidence of peri-apical pathology was noted. Placement of full metal onlays was planned on the mandibular left first and second molars with the one-step Dahl approach. While the mandibular left first molar with mild-moderate tooth surface loss was routinely monitored without active restorative procedure as no discomfort was reported and causes of tooth surface loss were well identified and controlled. Study casts were mounted on a semi-adjustable articulator (Denar® Mark II-Whip Mix, Louisville, KY) in a centric relation for assessment. The incisal pin of the articulator was raised 1.5 mm to provide an inter-occlusal space for the accommodation of metal crowns and establish the occlusal morphology on the right mandibular first and second molars [6]. A diagnostic wax-up was performed on duplicated study casts (Figure 1C).

**Figure 2:**
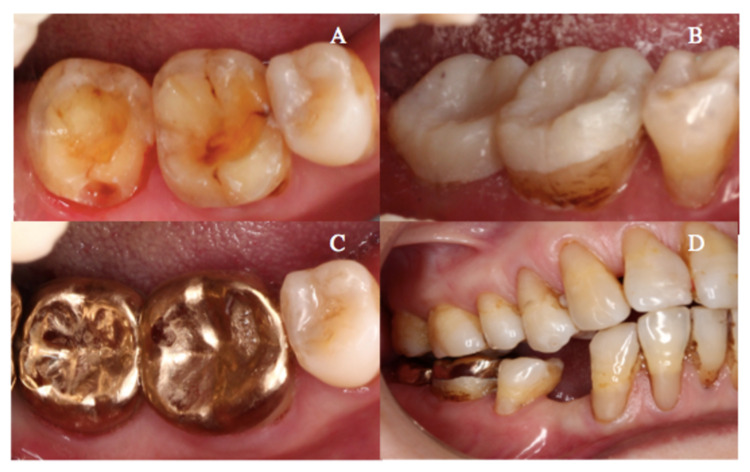
Tooth preparation, provisional onlays, and metal onlay cementation in the one-stage Dahl approach (A) Mandibular right first and second molars were prepared with minimal chamfer on axial walls. (B) Provisional crowns were cemented on the mandibular right first and second molars. (C) Occlusal view of the mandibular right first and second molars following cementation of gold onlays. (D) Right buccal view of the mandibular right first and second molars immediately after cementation of gold onlays.

The mandibular right first and second molars were prepared with minimal chamfer on the axial wall within the enamel (Figure 2A) The teeth surface was subsequently spot-etched with 35% phosphoric etchant (3M^TM^ Scotchbond Universal Etchant, Saint Paul, Minnesota) and temporalized with provisional onlays using flowable composite resin (Bisco Core-Flo™ DC, Chicago, Illinois) (Figure 2B). Type III gold onlays were heat-treated at 400°C for four minutes to improve the resin adhesion and cemented on the prepared teeth at 1.5 mm increased occlusal vertical dimension (Figures 2C-2D) as planned using resin cement (Panavia Ex, Kuraray, Japan).

**Figure 3:**
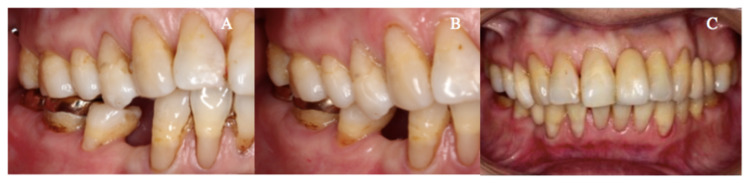
Progression of complete occlusal re-establishment and postoperative photograph (A) Occlusal contact re-establishment was in progress at the first month after onlay cementation on the mandibular right first and second molars. (B) Right buccal view of complete occlusal contact re-establishment during the second-month follow-up. (j) Postoperative photograph with complete occlusal re-establishment and replacement of missing maxillary left central incisor, first and second premolars, as well as mandibular right first premolar with resin-bonded bridges.

The patient was subsequently reviewed on a monthly basis (Figures 3A-3B). Re-establishment of complete occlusal contact was noted in the second month after gold onlay cementation (Figure 3B). All the maxillary and mandibular missing teeth were then replaced with resin-bonded bridges. Upon treatment completion, the patient was fully satisfied with her restored chewing function. Her one-year postoperative photograph is shown in Figure 3C.

Case report 2

A 58-year-old woman presented to the Restorative Specialist Clinic, The National University of Malaysia, complaining of sensitivity at a mandibular right tooth when consuming cold beverages. Tooth hypersensitivity in the area of complaint had caused chewing function impairment. She had hypertension and was on a regular dose of Atenolol 100 mg daily. Her favorite fruit was pineapple, which the patient admitted to taking a few times a week for the past few years. Informed consent was obtained from the patient before starting treatment.

**Figure 4:**
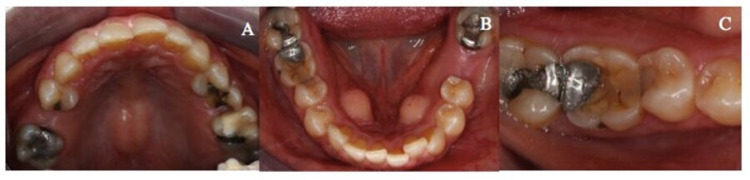
Preoperative photographs (A) Preoperative maxillary occlusal view. (B) Preoperative mandibular occlusal view. (C) Right mandibular first molar presented with moderate tooth wear with amalgam stood proud from the surrounding tooth structure.

No abnormality was detected upon the extra-oral examination. The intra-oral examination revealed maxillary and mandibular partially dentate with missing maxillary right and mandibular left first molars (Figures 4A-4B). The mandibular right first molar was moderately restored with a cupping-out sign noted on its occlusal surface (Figure 4C). The amalgam on the disto-occlusal surface was intact but stood “proud” from the surrounding tooth structure (Figure 4C). The loss of inter-occlusal space between the mandibular right first molar and its opposing tooth was noted clinically. Pulp sensibility testing was positive with no radiographic evidence of secondary caries and peri-apical infection.

**Figure 5:**
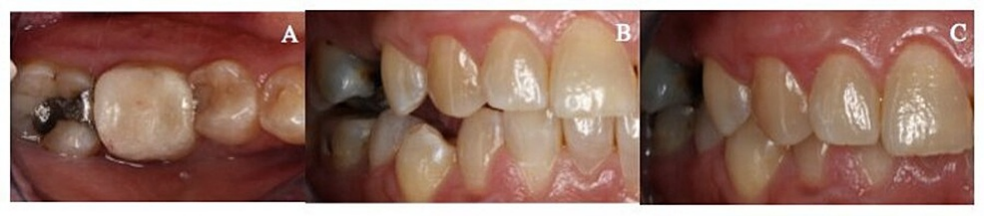
One-step Dahl procedure using direct composite resin (A) Placement of nano-hybrid composite resin at a thickness of 1.5 mm on the occlusal surface of the right mandibular first molar. (B) Occlusal contact re-establishment was in progress in the second month following composite build-up. (C) Occlusal contact was successfully re-established in the third month after composite build-up.

Study casts were mounted on a semi-adjustable articulator (Denar® Mark II-Whip Mix) in a centric relation based on the facebow record. A diagnostic wax-up was performed at the mandibular right first molar at an increased occlusal vertical dimension of 1.5 mm after careful assessment of the study casts. Active restorative treatment was initiated after controlling the root cause. To increase the bonding surface area, amalgam on the disto-occlusal surface of the mandibular right first molar was removed. Enamel layers on the distal cavity and occlusal surface of the tooth were then selectively etched with 35% phosphoric etchant (3M^TM ^Scotchbond Universal Etchant) to minimize the possibility of tooth hypersensitivity, followed by application of prime and bond (3M^TM^ ESPE Single Bond Universal Adhesive) on all distal cavity and occlusal surfaces prior to incremental placement of nano-hybrid composite resin (3M^TM^ Filtek Z350XT Universal Restorative) up to 1.5 mm thickness (Figure 5A) [9]. The incremental composite build-up was achieved by using a stable and rigid polyvinylsiloxane matrix guide (3M^TM^ Express XT Putty Vinyl Polysiloxane Impression Materials) derived from the lingual aspect of the diagnostic wax-up. Intolerance to the Dahl fixed appliance was reported in the first week after composite placement but the issue was subsequently resolved. The progress of occlusal contact re-establishment was regularly monitored (Figure 5B), and it was achieved in the third month after composite build-up (Figure 5C). No sign of composite fracture or dislodgement was noticed during the observation period. The patient was happy with the treatment as her chewing function was improved with the absence of tooth hypersensitivity.

## Discussion

Erosive tooth wear is not uncommon as a result of dietary habits [10]. Both the patients described here suffered moderate to advanced tooth wear, which was mainly localized on the right mandibular molars possibly due to frequent consumption of acidic food accompanied by a unilateral chewing habit. Active restorative interventions were necessary after addressing the root causes of the patients’ tooth wear, as their chewing functions were impaired due to symptoms of pain and discomfort.

In both cases, short clinical crown height coupled with limited inter-occlusal space made restorative management more challenging. To address these clinical challenges, the Dahl approach was adopted as the preservation of tooth structure is of prime concern. In comparison to the commonly used two-step Dahl procedure, the one-step Dahl procedure used in the two cases above is simpler, as no interim appliance is needed to create an inter-occlusal space. The definitive restorations that were placed on the worn teeth in supra-occlusion enabled occlusal contacts to be re-established via subsequent intrusion of the affected posterior worn teeth and eruption of the remaining teeth [9]. 

Patients’ intolerance to fixed appliances can be a potential problem in the one-step Dahl procedure. However, both our patients could fully tolerate such appliances one week later [7]. Besides, both patients were not affected by temporomandibular joint dysfunction (TMJD), which may uncommonly complicate this procedure [11].

With good adhesive material, the usage of fixed Dahl appliances made from direct composite resin or metal onlays on an affected posterior worn tooth can achieve a good outcome with a careful selection of cases. In comparison to zirconia-based restorations, the aforementioned materials provide better bonding strength and are kind to opposing natural dentition [12-13].

For cases with more than one-third tooth structure loss as shown in the first case, the use of adhesive metal onlay (type III gold alloys) has an advantage over direct composite resin, as it helps overcome issues in polymerization shrinkage stress, which may potentially cause marginal microleakage. Type III gold alloys, which contain 70% gold, are suitable to be used on high-stress areas due to the increased hardness of the material. A predictable outcome can be achieved if structurally compromised teeth are conservatively prepared with all the margins kept supragingivally on the sound enamel [14]. To minimize further tooth structure loss, additional mechanical retentive features were not incorporated in the tooth preparation design for this case. Retention of gold onlays could be adequately achieved by using Panavia Ex cement (Kururay Dental, New York, NY), which contains 10-Methacryloyloxydecyl dihydrogen phosphate (10-MDP). 10-MDP helps increase the bonding strength between gold onlays and resin cement. Additionally, the bonding strength between the resin cement and gold onlays could be further improved via oxidation of gold onlays surface with heat treatments at 400°C for four minutes prior to onlay cementation [15].

In contrast, an aesthetically pleasing direct composite resin is an alternative option as a definitive restoration in the Dahl approach to effectively treat an erosive molar tooth with an adequate remaining amount of enamel as in the second case. The presence of an enamel rim is one of the important prerequisites to achieve predictable bonding strength between enamel and composite resin [16]. Enhancement of bonding strength can be further attained by increasing the surface area for bonding via enamel beveling [17] and the replacement of existing intact amalgam. Bulk fracture of composite resin is a common risk when it is placed on posterior dentition in supra-occlusion. However, this risk could be mitigated by using nano-hybrid composite resin, which could withstand higher functional occlusal loads [2]. A 1.5 mm-thick composite resin, which was incrementally placed in supra-occlusion helped minimize the risk of polymerization shrinkage that may lead to adhesive and cohesive failure [18]. Considerable wear of direct composite resin is a common issue but it occurs at a slower rate for nano-hybrid composites [19]. This has been further supported by a study by Van Dijken, which reported promising clinical longevity of direct composite onlays with a lower occlusal wear rate of 4.2% at 11-year follow-up [18]. Nevertheless, the material can be easily added on if the wear occurs subsequently following the completion of Dahl treatment.

The occlusal surface of posterior fixed appliances should be designed in shallow cusps to allow compressive forces to transmit along the long axis of the treated tooth within periodontal ligament spaces. This would limit excessive lateral forces during excursive mandibular movement [20], which could cause restoration failure. Patients without a history of para-function habits would predict a favorable outcome of the Dahl concept in the management of posterior erosive tooth wear.

Both our patients achieved complete occlusal contact re-establishment within three months time as compared to the mean duration of six months in a previous study using removable appliances that require high patient compliance [7]. Both patients were continuously monitored up to one year after complete occlusal contact re-establishment. The chewing function for both our patients was subsequently fully restored following occlusal contact re-establishment and replacement of the missing teeth.

## Conclusions

With comprehensive clinical examinations, careful case selection and treatment planning, the one-step Dahl approach can be less invasive as compared to conventional means in the management of a localized posterior worn tooth complicated by limited inter-occlusal space.
